# Supplementary material for: Super-resolution analysis of PACSIN2 and EHD2 at caveolae
Source: PLoS One. 2022 Jul 14;17(7):e0271003. doi: 10.1371/journal.pone.0271003 (PMC9282494; doi:10.1371/journal.pone.0271003)
Supplement: S1 Fig — The average number of caveolin-1 blobs per observation, the XY standard deviation, and the Z standard deviation for each class and each combination of antibodies are shown. The dot represents an average from an observation, which typically contained one cell. N = 6–10 observations for each combination of antibodies. The combinations of antibodies are as follows: a: Caveolin-1 (7C8) + Caveolin-1 (3238), b: Caveolin-1 (3238) + EHD2 (G-3), c: Caveolin-1 (7C8) + EHD2 (11440-1-AP), d: Caveolin-1 (3238) + PACSIN2 (SAB-1402538), and e: Caveolin-1 (7C8) + PACSIN2 (Senju). (PDF) [file pone.0271003.s001.pdf]

a: Caveolin-1 (3238) (Caveolin-1 (7C8) + Caveolin-1 (3238))
b: Caveolin-1 (3238) (EHD2 (G-3) + Caveolin-1 (3238))
c: Caveolin-1 (7C8) (EHD2 (11440-1-AP) + Caveolin-1 (7C8))
d: Caveolin-1 (3238) (PACSIN2 (SAB-1402538) + Caveolin-1 (3238))
e: Caveolin-1 (7C8) (PACSIN2 (Senju) + Caveolin-1 (7C8))

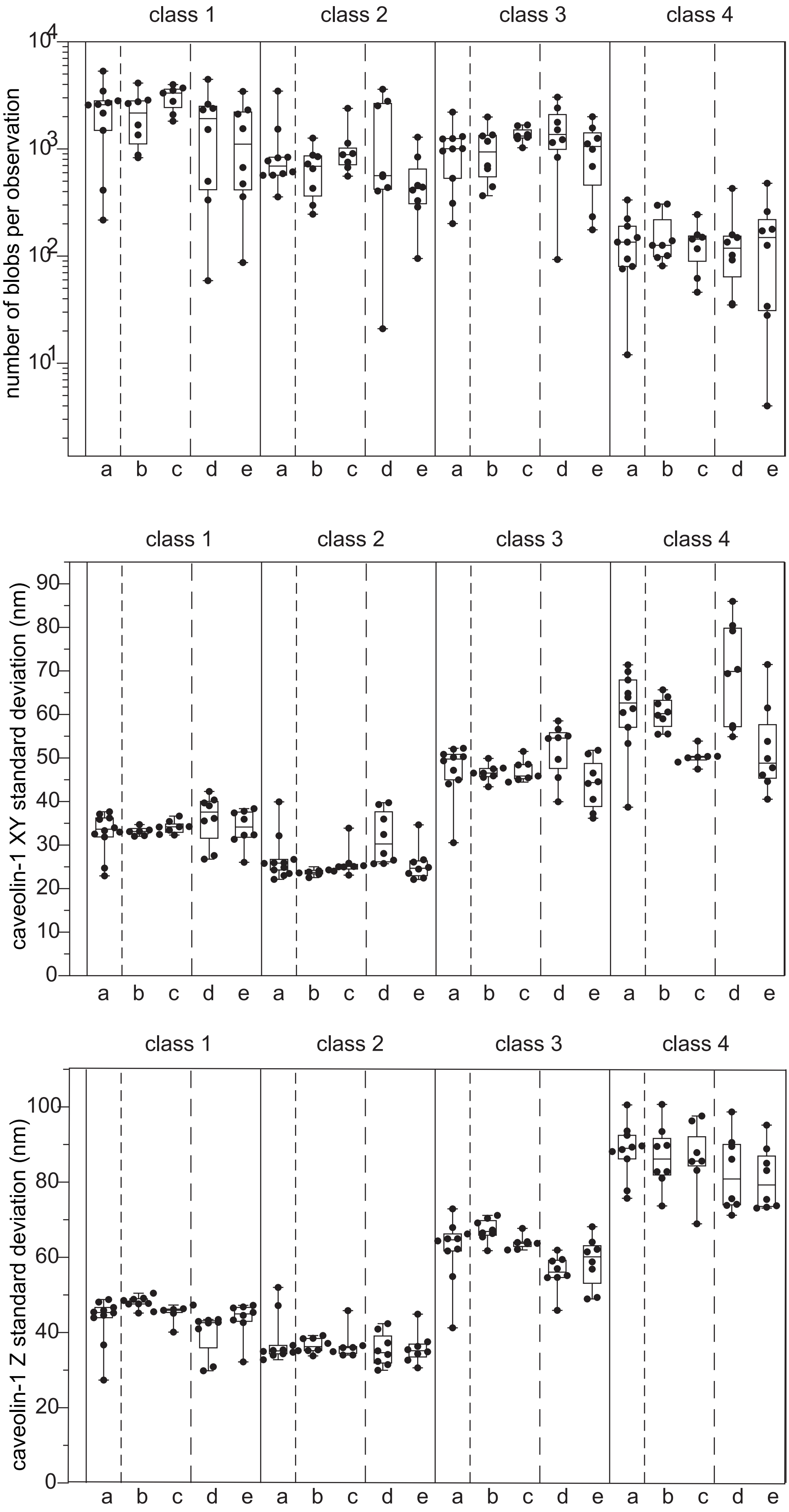

Figure S1
