# Supplementary figures and images for: Super-resolution analysis of PACSIN2 and EHD2 at caveolae
Source: PLoS One. 2022 Jul 14;17(7):e0271003. doi: 10.1371/journal.pone.0271003 (PMC9282494; doi:10.1371/journal.pone.0271003)

- wo: Caveolin-1 blobs without the colocalization

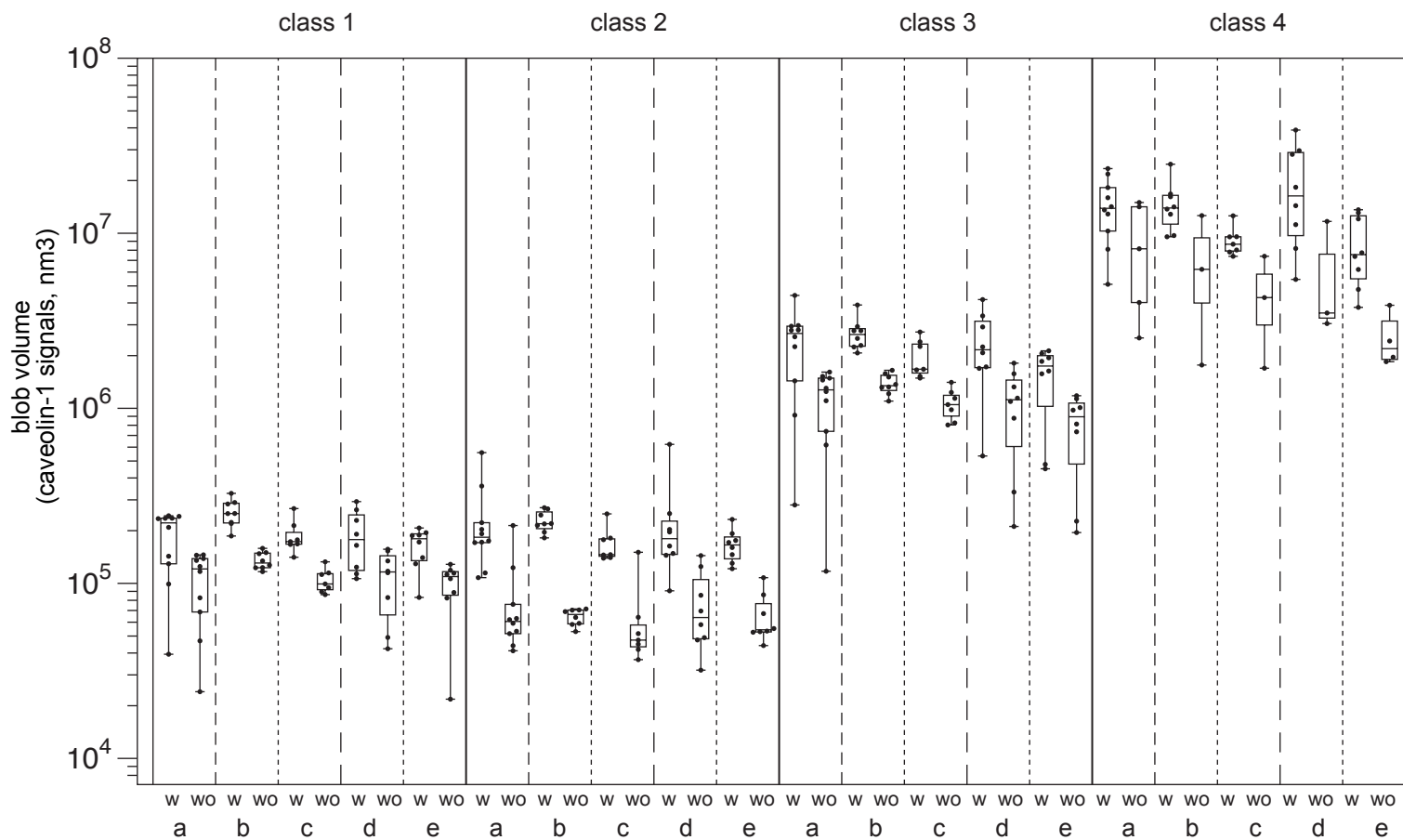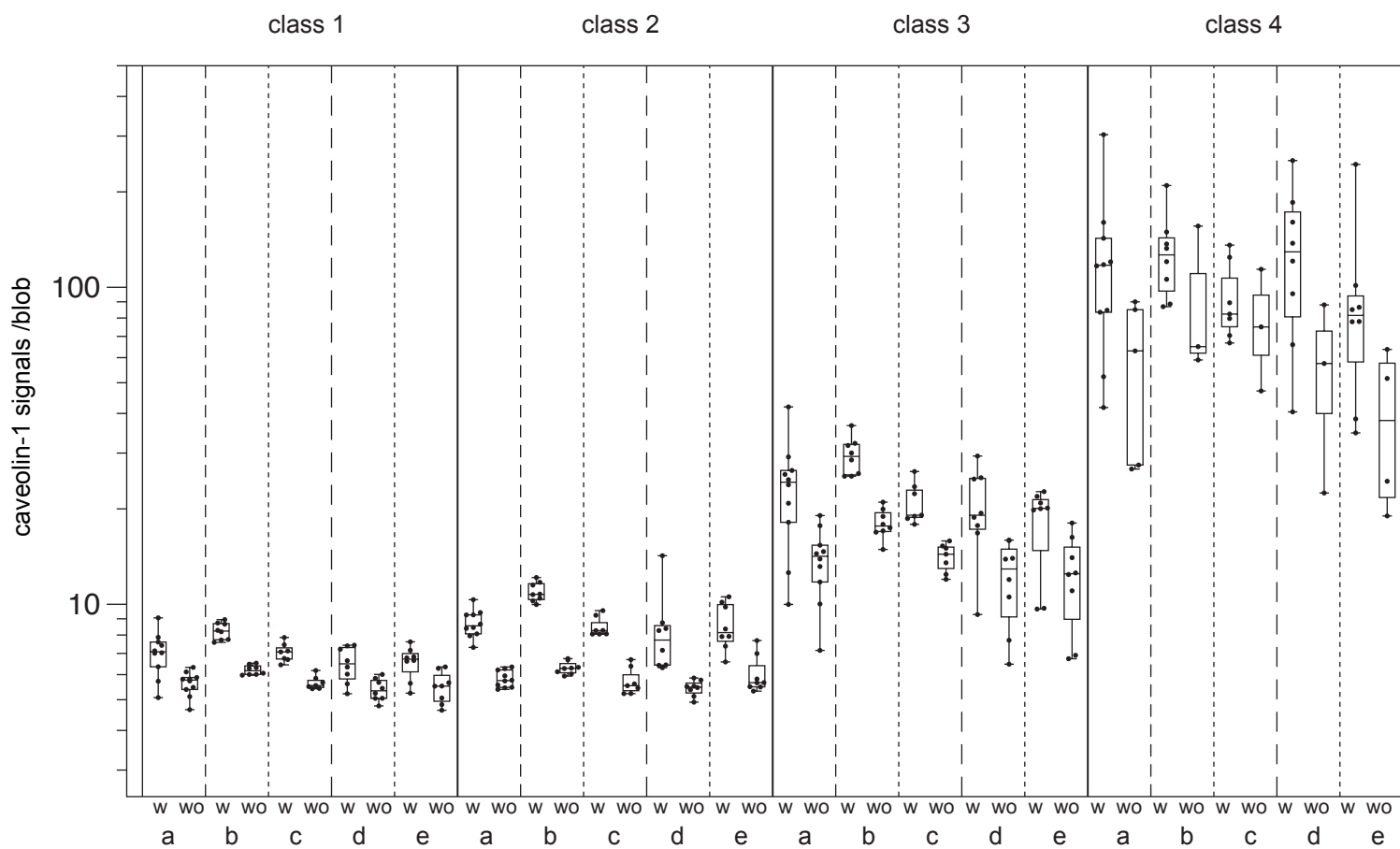

Figure S2

Supplement: S2 Fig — The volume of a caveolin-1 blob and the average number of caveolin-1 signals per blob per observation, obtained for each class for each combination of antibodies, shown with or without the colocalization of the two stains. The dot represents an average from an observation, which typically contained one cell. N = 6–10 observations for each combination of antibodies described in S1 Fig. w: Caveolin-1 blobs with the colocalization; wo: Caveolin-1 blobs without the colocalization. (PDF) [file pone.0271003.s002.pdf]
