# Supplementary material for: Super-resolution analysis of PACSIN2 and EHD2 at caveolae
Source: PLoS One. 2022 Jul 14;17(7):e0271003. doi: 10.1371/journal.pone.0271003 (PMC9282494; doi:10.1371/journal.pone.0271003)
Supplement: Attachment — Submitted filename: ReviwerComments220601v2.docx [file pone.0271003.s006.docx]

*Reviewer #1: The authors have now significantly improved their manuscript. They have improved both the figures and the text and proof-read their MS.*
A) We would like to appreciate your careful comments on the improvement of our manuscript.

*Minor points:
the authors must simplify the presentation of Figure 3D, this panel is too complicated for the reader and also modify Figure 3E schematic as caveolae are never forming tubule-like structures but they have an omega shape.*

A) We improved the Figure 3D for simplicity and we modified Figure 3E into the omega shaped one.

*Reviewer #2: The manuscript has greatly improved.
However, there are some issues that need to be discussed and corrected before publication.*
A) We would like to appreciate your careful comments on the improvement of our manuscript. We modified it further according to your comments. *1) There are some incorrect uses of references in the introduction.
“there are four cavin isoforms, and they are essential for caveolae”. Ref 11, only covers CAVIN1, the three original “cavin family papers” should be referenced here to be precise doi: 10.1083/jcb.200903053,doi: 10.1038/ncb1887, doi: 10.1038/emboj.2009.46. In addition, Professor Pilch’s original papers on the discovery of CAVIN1 functional importance are not cited, the authors could also consider to include these for the statement on CAVIN1.*

*Ref 26 is the original of EHD2 localisation to caveolae, so the authors should also include this when being discussed.*
A) We appreciate for pointing these important papers. We cited these papers (Bastiani et al., 2009; Hansen et al., 2009; Liu and Pilch, 2008; McMahon et al., 2009) for CAVINs. The ref26 (now ref 32) is also at line 63 for the discussion of EHD2 localization.

*2) Needs detailed discussion on the limitations of using primary/secondary Abs to study the fine scale localisation of components within structures as small as caveolae.*

A) According to this suggestion, we added several sentences.

Line 132-138: Therefore, the number of molecules to be detected is related to the volume of the blobs and the number of observed signals, suggesting that the class 1 and 2 blobs might have an insufficient number of molecules for colocalization detection or might not have colocalization. Such difficulty in analyzing small structures would probably result from the antibody labeling, where only a small fraction of the proteins can be visualized, as has been shown that 1-8 signals per caveola by electron microscopic analysis by using various caveolin-1 antibodies (Fujimoto et al., 2000). *3) Are student T-tests (and what type was used?) appropriate for these type of analysis? The authors need to explain the justification for this. The use of correct statistical test is critical.*
A) The comparison can be justified only between the caveolin-1 (7C8) antibody-treated groups for the PACSIN2 and EHD2 localizations by the two different antibodies. Therefore, we believe a two-tailed t-test is suitable. However, we also performed one-way ANOVA with post-hoc Holm-Bonferroni analysis, and p values are replaced.

*Minor comments

Line 42 “quite similar” is a subjective term, and the authors could consider changing this ot be more specific.*

A) It was rephrased to “almost identical” with citation.

Line 42: Caveolin exists as three isoforms, and the caveolin-1 and caveolin-3 amino acid sequences are almost identical (Tang et al., 1996; Way and Parton, 1995).
 *Line 81 Change “However, Pacsin2 was” to “However, Pacsin2 is”*
A) This part is the summary of the results of this paper, and we would like to keep “was” here.

*Line 87, no mention of what type of cells, this is first introduced in line 89, introduction should be in line 87.*
A) We corrected it accordingly.

Line 89: First, we observed the single-molecule localizations in antibody-stained HeLa cells.

*Line 115, ref style for Khater, 2018 is not correct.*

A) We formatted it.

Bastiani, M., Liu, L., Hill, M.M., Jedrychowski, M.P., Nixon, S.J., Lo, H.P., Abankwa, D., Luetterforst, R., Fernandez-Rojo, M., Breen, M.R., et al. (2009). MURC/Cavin-4 and cavin family members form tissue-specific caveolar complexes. J Cell Biol *185*, 1259-1273. 10.1083/jcb.200903053.

Fujimoto, T., Kogo, H., Nomura, R., and Une, T. (2000). Isoforms of caveolin-1 and caveolar structure. Journal of cell science *113*, 3509-3517.

Hansen, C.G., Bright, N.A., Howard, G., and Nichols, B.J. (2009). SDPR induces membrane curvature and functions in the formation of caveolae. Nat Cell Biol *11*, 807-814. 10.1038/ncb1887.

Liu, L., and Pilch, P.F. (2008). A critical role of cavin (polymerase I and transcript release factor) in caveolae formation and organization. J Biol Chem *283*, 4314-4322. 10.1074/jbc.M707890200.

McMahon, K.A., Zajicek, H., Li, W.P., Peyton, M.J., Minna, J.D., Hernandez, V.J., Luby-Phelps, K., and Anderson, R.G. (2009). SRBC/cavin-3 is a caveolin adapter protein that regulates caveolae function. Embo j *28*, 1001-1015. 10.1038/emboj.2009.46.

Tang, Z., Scherer, P.E., Okamoto, T., Song, K., Chu, C., Kohtz, D.S., Nishimoto, I., Lodish, H.F., and Lisanti, M.P. (1996). Molecular cloning of caveolin-3, a novel member of the caveolin gene family expressed predominantly in muscle. J Biol Chem *271*, 2255-2261. 10.1074/jbc.271.4.2255.

Way, M., and Parton, R.G. (1995). M-caveolin, a muscle-specific caveolin-related protein. FEBS Lett *376*, 108-112. 10.1016/0014-5793(95)01256-7.
